# Disseminated Nocardiosis in renal transplant recipient under therapy for pulmonary tuberculosis: a case report

**DOI:** 10.1186/s13104-017-2408-0

**Published:** 2017-02-03

**Authors:** Priyatam Khadka, Ramesh Bahadur Basnet, Pratap Khadka, Dibya Singh Shah, Bharat Mani Pokhrel, Basista Parsad Rijal, Jeevan Bahadur Sherchand

**Affiliations:** 1 Tribhuvan University Teaching Hospital, Kathmandu, Nepal; 2 Trichandra Multiple Campus, Tribhuvan University, Ghantaghar, Kathmandu, Nepal; 3 Central Department Tribhuvan University, Ghantaghar, Kathmandu, Nepal; 4 Deerwalk Services Pvt. Ltd, Kathmandu, Nepal; 5 Department of Nephrology, Tribhuvan University Teaching Hospital, Kathmandu, Nepal; 6 Institute of Medicine, Kathmandu, Nepal; 7 Department of Microbiology, Tribhuvan University Teaching Hospital, Kathmandu, Nepal

**Keywords:** Disseminated Nocardiosis, Pulmonary tuberculosis, Renal transplant, Cerebral abscesses, Immune suppression

## Abstract

**Background:**

Nocardiosis is an opportunistic infection in a patient with underlying immune suppression and organ transplant. Clinical syndromes are varied and ranges from pulmonary, disseminated, cutaneous along with central nervous system involvement.

**Case presentation:**

Herein, we report a rare case of disseminated pulmonary nocardiosis with cerebral manifestation in a 66 year-old-Nepali farmer; with a history of renal transplantation and undergoing therapy for pulmonary tuberculosis. Radiographic imaging revealed multiple opacities of varying sizes in bilateral lung field mediastinal, retroperitoneal lymphadenopathy, and ill-defined lesion with surrounding edema seen in left occipitoparietal region of brain. Bacteriological assessments of bronchoalveolar lavage and purulent fluid extracted intra-operatively from the lesion confirmed the case as Nocardiosis.

**Conclusion:**

Disseminated Pulmonary nocardiosis with central nervous system involvement carries a poor prognosis. However, early diagnosis of the case, the administration of appropriate antibiotic, stereotactic aspiration alone or craniotomy has a successful outcomes even in a post renal transplant patient treated with anti tuberculosis treatment.

## Background

Nocardiosis, is an opportunistic infection particularly in a patient with underlying immune suppression. The clinical syndromes varies and range from pulmonary, disseminated, cutaneous form involving eyes, kidneys, skin, bone and CNS. The clinical manifestations may be subtle with the radiological findings mimicking other entities such as malignancy. The organism requires multiple days to grow in culture, thus detection and early treatment may be delayed [1]. The lungs are presumed as primary site of infection (60–80% of cases) and brain abscess is, by far, the most common complication conferring poor prognosis [2, 3]. Although, Nocardial cerebral abscesses account for only 1 to 2% of all cerebral abscesses the mortality rate due to it is considerably higher accounting for, 55 and 20% in immunocompromised and immunocompetent patients, respectively [4]. Treatment is based on stereotaxic aspiration or surgical resection, and a course of antibiotics therapy for several months. Since, It has been estimated that the prevalence of Nocardiosis and concomitant infection with tuberculosis is more than 6.25%, early detection can be a lifesaving approaches [5].

We present a case of disseminated nocardiosis in renal transplant recipient who presented with pulmonary and cerebral manifestation. The stereotaxic aspiration or surgical resection, and antibiotics therapy (trimethoprim/sulfamethoxazole) for several months was found to be crucial for prognosis.

## Case presentation

A 66-year-old Nepali farmer from Kathmandu with a history of renal transplant in July 2013 undergoing medication for pulmonary tuberculosis since 4th June, 2014.The person was habitual of smoking, chewing tobacco and cannabis, with hypertensive past history. After 4 month of ATT the patient acquired perinephric haemotoma which was drained out twice with pig tail drainage. Nearly, after 2 month (January 2015) the patient was admitted in TUTH under Nephrology unit with a chief complain of altered sensorium, hyponatremia and twitching of face. Following the admission, pyrazinamide was stopped from ATT until symptomatic improvement of the clinical case and re-added after a month following the Gene Xpert report. Tacrolimus level was assessed as 6.5 micro gm/dl with normal PBS, RFTs, and LFTs and the patient was discharged after 9 days. Six months later on June 1st, 2015, the patient was readmitted after the clinical findings of right UMN Facial palsy and slight pronator drift. A chief complain of chest pain, coughing and evening rise of fever was revealed by the patient prior to hospital re-admission. The physical examination revealed no limbs swelling, palpitations and loss of consciousness. No lymphadenopathy and organomegally was noted.

Contrast Enhanced Computed tomography of chest and abdomen showed multiple opacities of varying sizes in bilateral lung field mediastiral and retroperitoneal lymphadenopathy. Hyperdense area with enhancing wall in subcutaneous plane and muscular plane in abdomen and pelvis was observed.

**Fig. 1:**
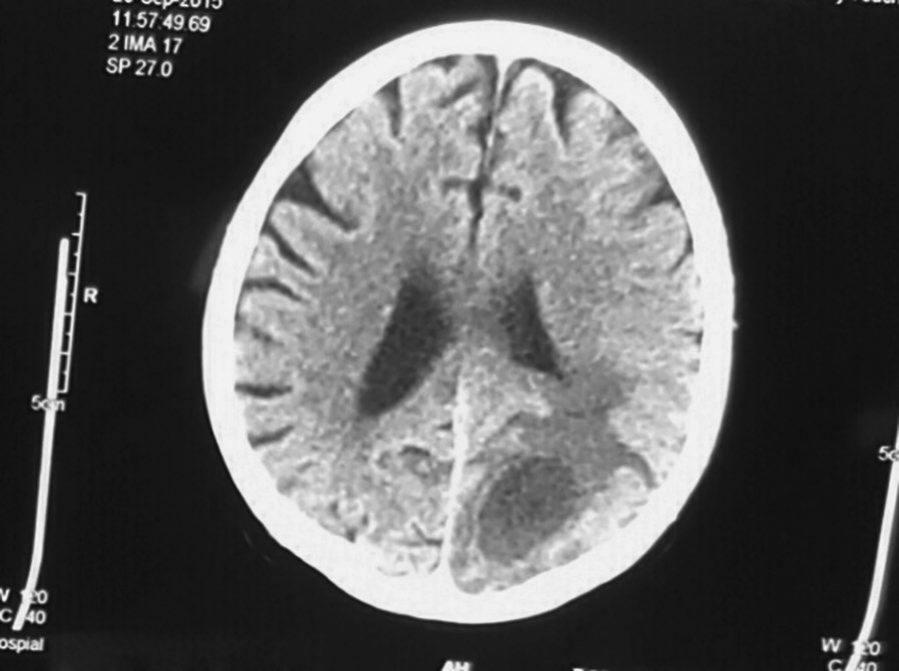
Head Computed tomography scan: ill-defined lesion with surrounding edema seen in *left* occipitoparietal region

**Fig. 2:**
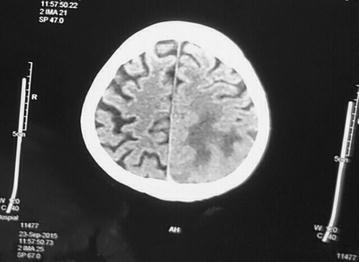
Head Computed tomography scan: ill-defined lesion with surrounding edema seen in *left* occipitoparietal region

Computed tomography scan of brain revealed an ill-defined lesion with surrounding edema in left occipito-parietal region, low grade glioma, granulomatous lesion, possibly tuberculoma (Figs. 1, 2). Differential diagnosis of abscess and contrast was suggested.

**Fig. 3:**
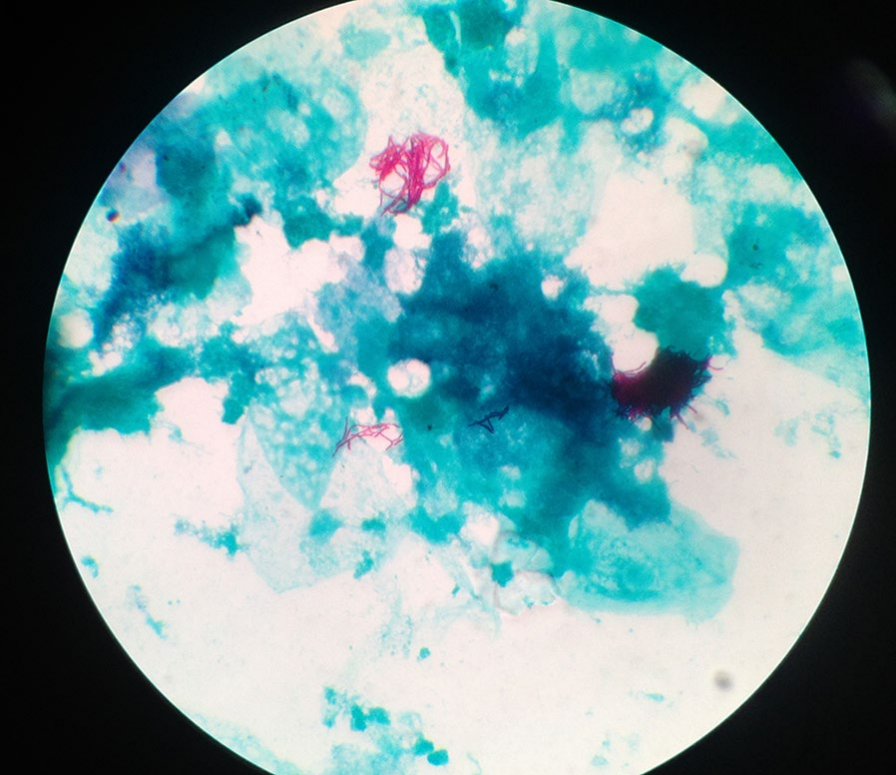
AFB staining: partially acid fast branching rod suggestive *Nocardia species* on modified. Kinyounstain (1000×  orginalmagnification)

**Fig. 4:**
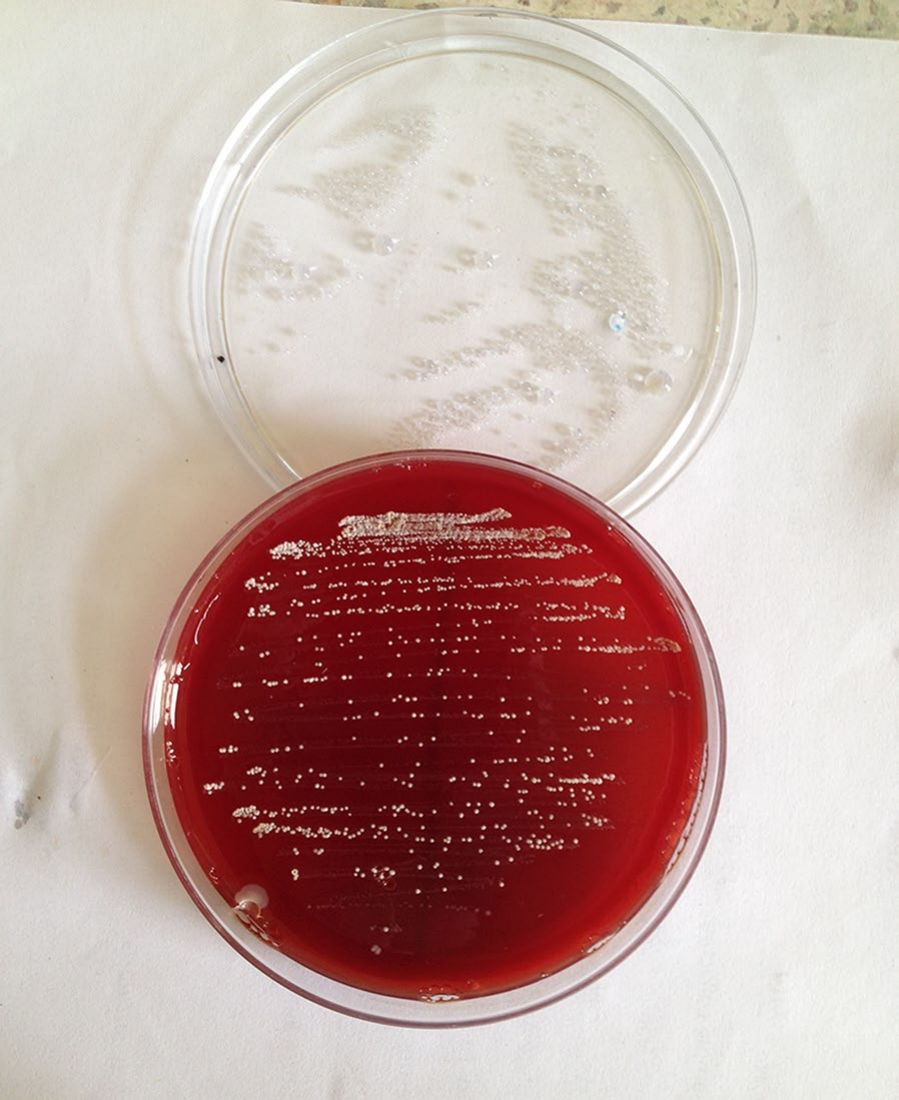
Colonial morphology of *Nocardia species* on Blood Agar:Whitish chalky adherent colonies of Nocardia species

**Fig. 5:**
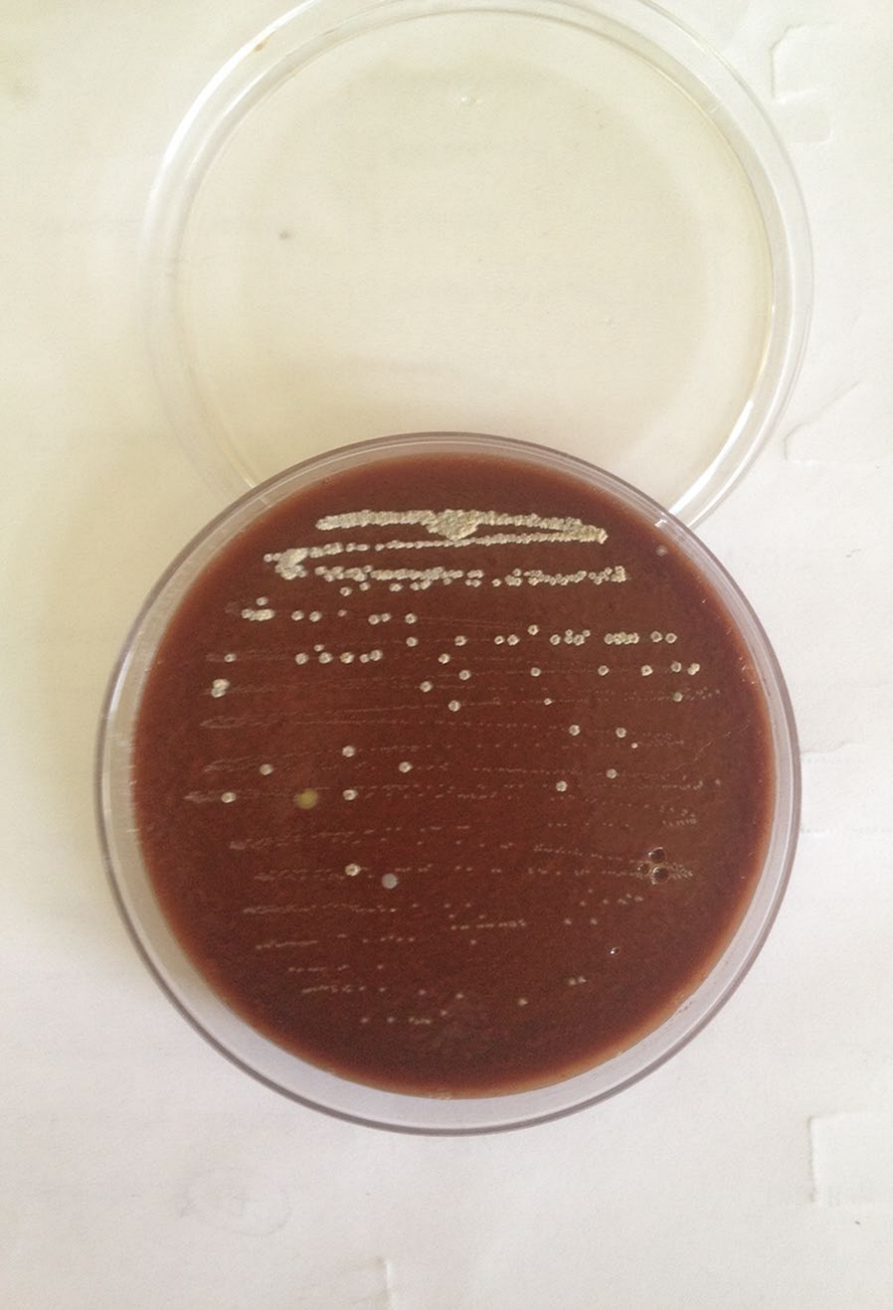
Colonial morphology of *Nocardia species* on Chocolate Agar:Whitesh chalky adherent colonies of Nocardia species

**Fig. 6:**
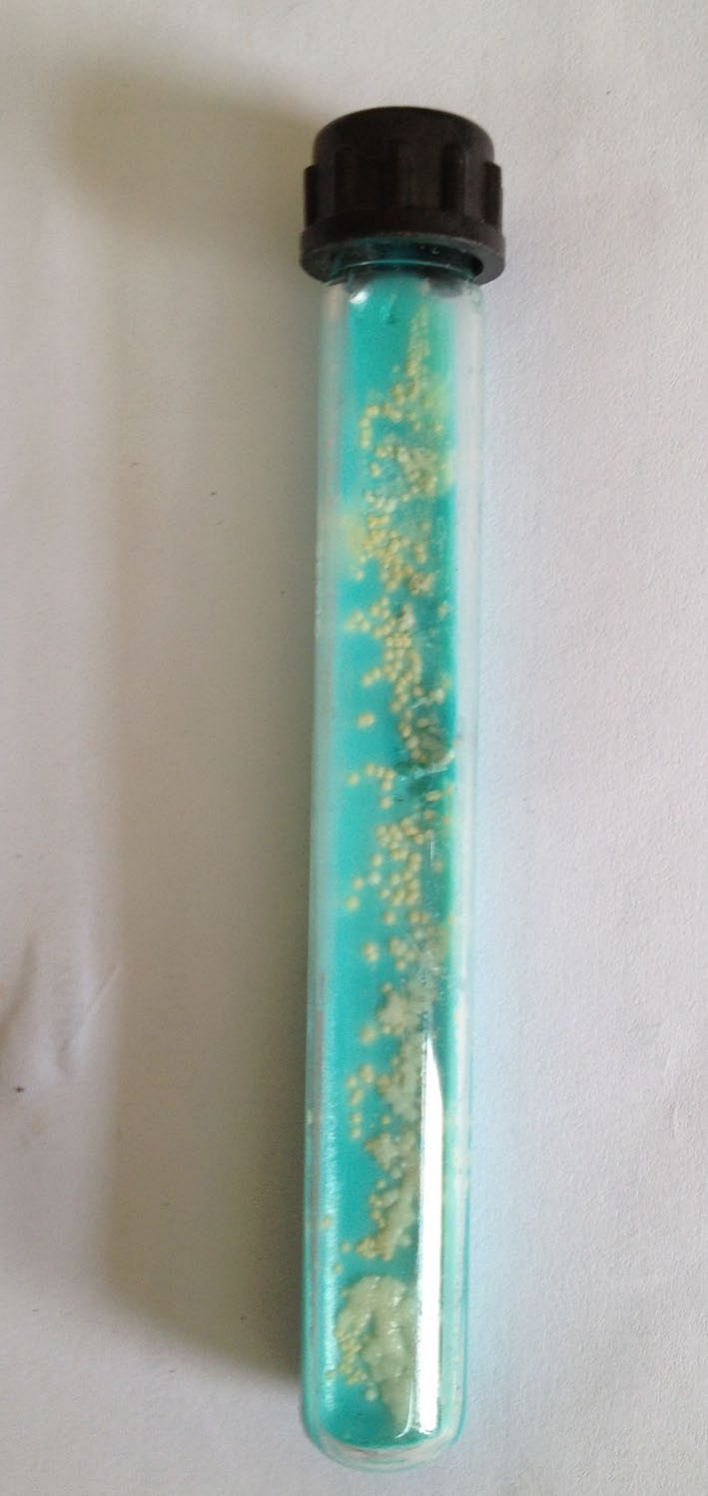
Colonial morphology of *Nocardia species* on Lowenstein–Jensen media:Whitesh chalky adherent colonies of Nocardia species

**Fig. 7:**
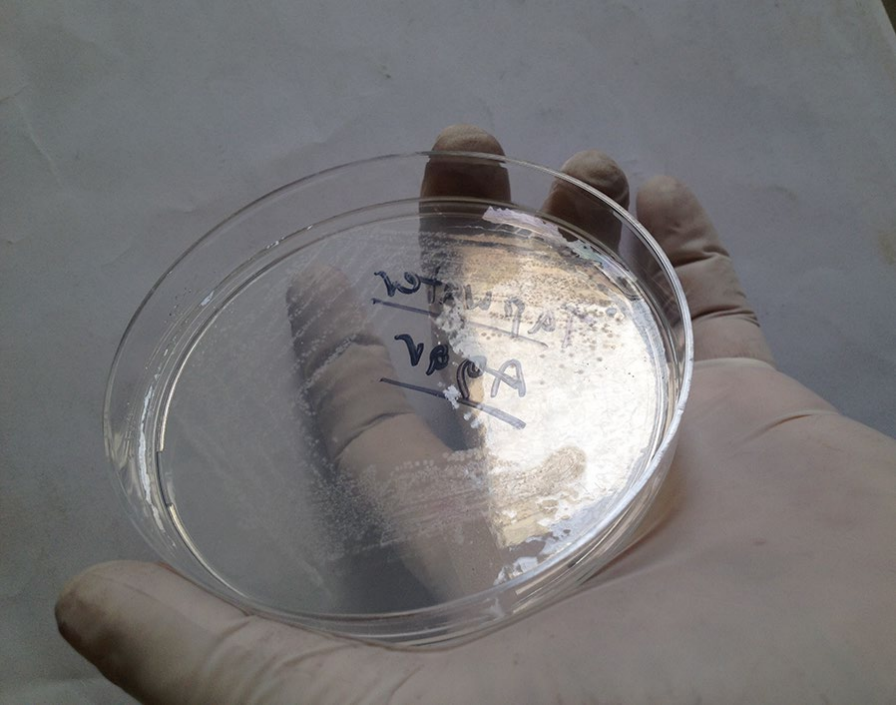
Colonial morphology of *Nocardia species* on Tapwater agar:Tap water agar showing areal hyphae

Presumptive identification was done with laboratory examination involving staining of broncho- alveolar lavage and purulent fluid from the lesion which was extracted intra-operatively. The laboratory findings revealed gram variable, acid fast branching rod suggestive *Nocardia species* (Fig. 3). Furthermore, upon aerobic culture of broncho-aleveolar lavage and purulent fluid from the lesion, chalky white adherent colonies were seen after 72 h of incubation with molar tooth appearance on Blood agar, Chocolate agar, LJ media and areal hyphae was observed on tap water agar (Figs. 4, 5, 6, 7) as identified through biochemical tests and results of antibiotic susceptibility tested carried out in compliance with the Manual of Clinical Microbiology and guidelines [6]. However, culture was negative for the fungal elements and blood culture was sterile. CSF examination including culture and assay for malignant cell was found to be negative.

**Fig. 8:**
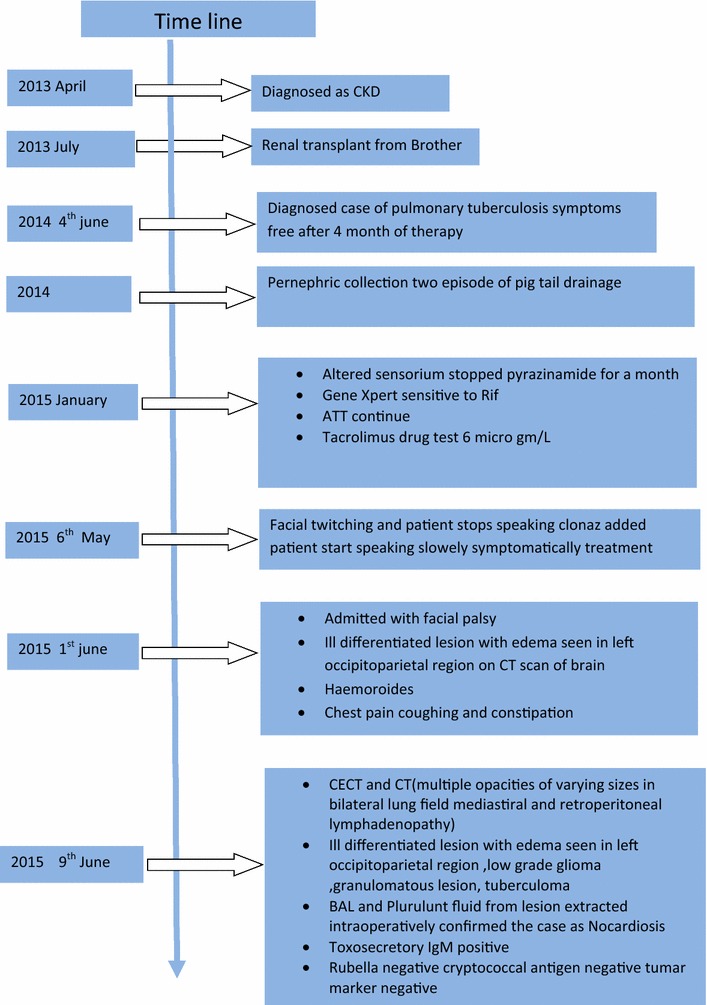
Timeline

Laboratory investigations of blood demonstrated neutrophil leukocytosis (1250), reduced hemoglobin (10 g/dl), C-reactive protein (positive), Rubella and CMV (Negative: ELISA), CMV retinitis (negative), Cryptococcal antigen(negative), Toxosecretory IgM (positive: ELISA). However the serological test for, Toxoplasma-specific IgM was later considered to be not infected. The patient’s blood was redrawn two weeks after the first and second tested together with the first specimen. Latter test revealed the patient to be negative for Toxoplasma-specific IgG and positive for toxoplasma-specific IgM confirming the earlier result of positive ELISA for Toxosecretory IgM to be false positive [8]. RFTs (urea, creatinine, electrolytes) ranges were normal and Tumor markers within normal limits (PSA, CEA,CA19.9).Urinary albumin(1+) was seen on routine urine examination. In view of examination and investigations, a diagnosis of disseminated pulmonary nocardiosis with CNS manifestations was made on post renal transplant patient under therapy for pulmonary tuberculosis. Stereotactic aspiration and craniotomy was performed for clinical management of the case and was treated with BACTRIM-DS (PO × BD), 2 double strength tablets each containing 800 mg sulfamethoxazole and 160 mg trimethoprim were prescribed. The patient recovered well after operation without neurological deficits, and then was discharged with prescription of 3 months antibiotic therapy to follow. The same medication was continued for 12 months as the patient underwent progressive changes and no relapse was noted with his transplant function observed in a good state. Given the progressive recovery of the patient without any symptoms that would alert the presence of disorder additional or alternative parenteral antimicrobial therapies of carbapenems (imipenem or meropenem, but not ertapenem), third generation cephalosporins (cefotaxime or ceftriaxone), and amikacin, alone or in combination were thought unnecessary to include in regimen though recommended by some author. Duration of antimicrobial therapy was further extended to minimize risk of late relapse. He is under regular follow-up since then and we found him asymptomatic, with limited side effects of prolonged antimicrobial therapy. Figure 8 graphically shows a time line of clinical history of patient and diagnostic approaches.

## Discussion

Post-transplantation TB is predominantly the result of reactivation of an earlier quiescent TB focus, previous TB anamnesis and family history, nosocomial acquisition or donor transmission, extended time spent on dialysis, prolonged pre-transplant hemodialysis, previous history of TB (9.5 to 13.5 %), Immunosuppressive drugs (steroids, mycophenolate mofetil, azathioprine, tacrolimus, antilymphocyte serum), diabetes and multiple episodes of acute rejection [7]. The incidence of post transplantation tuberculosis observed in developing countries is about 20 to 74 folds higher than that to the general population.

Apart from these, Anti-tuberculosis drugs have their own spectrum of hematological toxicity and blood cell abnormalities along with drugs induced syndromes (hemolytic anemia, methemoglobinemia, red cell aplasia, sideroblastic anemia, megaloblastic anemia, polycythemia and aplastic anemia) [8]. On the same basis, it raise concerns that the use of various antibacterial agents affect natural immune functions essential to the clearance of invading microorganisms, particularly in patients known to be at higher risk of acquiring secondary infections [9]. Nocardiosis is prior of such secondary infection in above presented circumstances.

*Nocardia* is classically described as gram-variable, aerobic, filamentous, branching, weakly acid-fast bacilli; in various sub-optimal growth conditions. *Nocardia* may appear gram-negative and acid-fast-stain negative with longer incubations period for growth. Hence, Identification of *Nocardia species* in the clinical laboratory is challenging and high index of suspicion should be maintained where the patient symptomatology and chronicity of the diseases suggests or point towards an alternative diagnosis of nocardiosis.

Pulmonary nocardiosis with CNS manifestations is by far the most common in patients with post-transplant immunosuppression and immune suppressive drugs (tacrolimus, mycophenolatemofetil, and prednisolone) as discussed in the case under current consideration.

Nocardiosis tends to behave as pyogenic bacteria, possibly metastasizes haematogenously into distant organs system (lungs, central nervous system, eyes, kidneys, skin, subcutaneous tissue and bone) resulting fatal outcomes. Pulmonary involvement is the most common nocardial infection characterized with alveolar or interstitial infiltrates, single or multiple nodules, along with or without cavitation [10]. Pulmonary nocardiosis is a well described infection with neoplastic disease, HIV infection, and those receiving treatments with corticosteroids or various chemotherapeutic agents.

Virulent *Nocardia* species inhibits neutrophilic killing by macrophage via high levels of enzymes catalase and superoxide dismutase, and can pass the through endothelial cells to invade the brain where it infects both microglia and astrocytes. The *Nocardia* species have a special tropism for the neural tissue and most common site for their dissemination is the brain. CNS *Nocardiosis* may progress or relapse despite the physician’s best efforts to control the infection through antimicrobial therapy, with cure rates in brain abscesses reported at 50% and mortality as high as 55% [1]. The Nocardial brain abscesses is extremely rare, to the best of our knowledge, it is the first case reported from Nepal.

Clinical symptoms and Radiological Imaging findings are non-specific for diagnosis of Nocardiosis, however it’s importance cannot outweighed for the differential diagnosis. The presumptive identification of our case was done via staining of broncho-alveolar lavage and purulent fluid from the lesion intra-operatively; which revealed gram variable, acid fast branching rod suggestive *Nocardia* species. Furthermore, on aerobic culture chalky white adherent colonies seen after 72 h of incubation which turns molar tooth appearance on further incubation. Later on the organism was isolated as *Nocardia asteroides* with biochemical interpretation and sensitivity pattern of antibiotics following the CLSI guidelines.

The treatment of choice recommended by multiple authors, for disseminated pulmonary nocardiosis with CNS manifestation is long term sulfonamide therapy alone or in combination with imipenem, meropenem, amikacin, ampicillin, 3rd generation cephalosporins, fluoroquinolones or minocycline [10]. Studies have demonstrated more than adequate penetration of antimicrobials into brain abscesses but differences in outcomes have based on surgical treatment, whether patients received stereotactic aspiration alone or craniotomy [1].

Nevertheless, the possibility of late recurrent nocardiosis could be pitfall to the successful outcomes. Henceforth, clinical management along with the consistent follow up is decisive. The recurrence of CNS nocardiosis is a multifactorial which depends on immune status of the patient, duration of antimicrobial prophylaxis after primary infection, the antimicrobial susceptibility profile of nocardial strains, and, potentially, the poor penetration of drugs to the CNS and so on [11]. It has been projected that the patient receiving TMP-SMX confer a relapse rate of about 13.6% (compared to 32% mortality rate and 16% relapse rate in patients who did not receive TMP-SMX) [11]. These are the facts that imperil for the consideration of clinical management in nocardiosis.

The burden and case related to disseminate nocardiosis in developing countries like Nepal is undetermined but speculated to be sporadic. The unfamiliarity with the case, nonspecific or lack of pathognomonic clinical presentation, diagnostic intricacies, and lack of systematic reporting preclude further work up for nocardiosis. Therefore, multidisciplinary approach, utilizing speciation and sensitivity testing as well as appropriate surgical treatment, is critical to early microbial control of cerebral nocardiosis [12, 13]. Furthermore, appropriate diagnostic strategies are needed because clinical manifestations may be subtle and the appearance on imaging may mimic other entities such as malignancy and hence would be a tool to preventing relapse cases.

## Conclusion

Since the clinical and radiological manifestations are nonspecific and micribiological diagnosis is often difficult (Fig. 8). Nocardiosis should be considered in differential diagnosis among immunocompromised individuals, and recognition of predisposing factors is decisive for the prognosis. Apart from these a high index of clinical suspicion together with close collaboration with microbiological laboratory allows for more accurate diagnosis to initiate appropriate therapy, hence to reduce mortality significantly.

## Abbreviations

ATT: anti tuberculosis treatment
BAL: broncho-alveolar lavage
CLSI: Clinical and Laboratory Standards Institute
CMV: cytomegalovirus
CNS: central nervous system
CEA: carcinoembryonic antigen
CECT: contrast enhanced computed tomography
CT: computed tomography
DLC: differential leucocytes count
ELISA: enzyme linked immuno sorbent assay
IgG: immunoglobulin G
IgM: immunoglobulin M
HIV: human immuno deficiency virus
LFT: liver function test
LJ: Lowenstein Jensen
PBS: peripheral blood smear
PSA: prostate-specific antigen
RFT: renal function test
TB: tuberculosis
TLC: total leucocytes count
TMP/SMX: trimethoprim/sulfamethoxazole
TU: Tribhuvan University
TUTH: Tribhuvan University Teaching Hospital
UMN: upper motor neuron
